# Roles of Myeloid and Lymphoid Cells in the Pathogenesis of Chronic Obstructive Pulmonary Disease

**DOI:** 10.3389/fimmu.2018.01431

**Published:** 2018-06-21

**Authors:** Ling Ni, Chen Dong

**Affiliations:** Institute for Immunology and School of Medicine, Tsinghua University, Beijing, China

**Keywords:** chronic obstructive pulmonary disease, immune system, immune cells, COPD pathogenesis, immune target

## Abstract

Chronic obstructive pulmonary disease (COPD) is currently the third largest cause of human mortality in the world after stroke and heart disease. COPD is characterized by sustained inflammation of the airways, leading to destruction of lung tissue and declining pulmonary function. The main risk factor is known to be cigarette smoke currently. However, the strategies for prevention and treatment have not altered significantly for many years. A growing body of evidences indicates that the immune system plays a pivotal role in the pathogenesis of COPD. The repeated and progressive activation of immune cells is at least in part the source of this chronic inflammation. In this review paper, we have conducted an extensive literature search of the studies of immune cells in COPD patients. The objective is to assess the contributions of different immune cell types, the imbalance of pro/anti-inflammatory immune cells, such as M1/M2 macrophages, Tc1/Tc10, and Th17/Treg, and their mediators in the peripheral blood as well as in the lung to the pathogenesis of COPD. Therefore, understanding their roles in COPD development will help us find the potential target to modify this disease. This review focuses predominantly on data derived from human studies but will refer to animal studies where they help understand the disease in humans.

## Introduction

Chronic obstructive pulmonary disease (COPD) is the third cause of human mortality worldwide after stroke and heart disease (1). It is estimated that approximately 20 million people in the United States alone are diagnosed with COPD (American Lung Association). Furthermore, COPD is a major risk factor for lung cancer (2). Around 50–80% of the patients diagnosed with lung cancer had pre-existing COPD (3, 4) and annual incidence of lung cancer arising from COPD has been reported to be 0.8–1.7% (5, 6). COPD, one of the few chronic diseases with rising prevalence has created a huge economic burden for both individuals and society. However, the strategies for prevention have not altered significantly for many years. Drug classes used in the treatment of COPD also have not changed dramatically, and none up to now have shown activity of modifying diseases. Currently, the first-line therapy for COPD patients is bronchodilators, although there is controversy about their high utility in this disease (7).

COPD is characterized as a progressive and irreversible decline in lung function caused by airflow obstruction, destruction of parenchyma, and emphysema (8). There is a chronic inflammation in the small airways of the lungs in those patients. Immunological processes have been implicated in the pathogenesis of COPD, since immunoferon-active principle (AM3), a natural glycoconjugate, which activates peripheral blood mononuclear cells (9) and enhances lymphocyte proliferation (10), can rescue the defective antimicrobial functions of the immune system effector cells of COPD patients, leading to improved life quality of COPD patients (11). The source of this chronic inflammation is attributed to the repeated and progressive activation of the innate and adaptive immune cells (12). The principle abnormalities in the airways are the presence of an inflammatory cellular infiltration and remodeling that thickens the airway wall (13). Attention has largely been focused on inhibition of recruitment and activation of these immune cells. Leukocyte migration is very important for maintaining host defense, but uncontrolled cellular infiltration into tissues can result in chronic inflammation. Moreover, although COPD affects patient lungs, it also produces significant systemic consequences. In this review, we have conducted an extensive literature search of the studies of immune cells in COPD patients. The objective is to assess the contributions of those immune cell types in the peripheral blood as well as in the lung during COPD progression and to explore the mechanisms by which those immune cells regulate the development of COPD.

## Innate Immune System

COPD is related to recruitment, infiltration and activation of inflammatory cells in the airways and pulmonary tissue. The innate immune response in the airways and lungs in COPD patients may be impaired (14). Several reports have showed that increased numbers of neutrophils and macrophages in the lungs have been detected in COPD patients. They are normal responders to tissue damage and oxidative stress induced by cigarette smoke. Furthermore, some other cell types in the innate system are also involved in COPD pathogenesis such as dendritic cells (DCs) and myeloid-derived suppressor cells (MDSCs).

### Neutrophil

Neutrophils represent a key component of the innate immune system. Neutrophils are associated with COPD severity and inflammation. They are key effector cells in COPD, while eosinophilia influx is typical to asthma. Recently, Walton et al. showed neutrophil function is dysregulated in COPD by their *in vitro* studies, which might contribute both to the destruction of lung parenchyma and to the poor responses seen in exacerbations caused by infections (15). Once there is an ongoing infection or inflammation, neutrophils leave the circulation and migrate to inflamed or infected sites. The airways of COPD patients are frequently colonized with bacteria and bacteria are major causes of COPD exacerbations. COPD patients with exacerbations tend to be more severe COPD and show larger neutrophil fraction in their induced sputum. An increase of neutrophil fraction in sputum cells is a significant predictor for COPD exacerbation (16). In peripheral blood, neutrophil to lymphocyte ratio is a useful systemic inflammatory response biomarker, and this ratio is also a straightforward and effective biomarker of COPD exacerbation, which may predict respiratory hospitalization in patients with COPD (17–19).

Neutrophils are the dominant circulating leukocyte. Bacterial extract, LPS, induces peripheral circulating neutrophils from COPD patients to secrete higher amounts of IL-8/CXCL8 (20, 21) as well as matrix metalloproteinase 9 (21). Moreover, several studies suggested that neutrophils from COPD patients display increased migration toward inflammatory stimuli (22, 23). In contrast, Sapey et al. (24) found that neutrophils from COPD patients (20 patients, mean age 60 years, 11 males, FEV1% 44) show higher migratory speed of movement in any direction but lower migratory accuracy toward inflammation, which is correlated to systemic neutrophil elastase (NE) activity. The discrepancy of accurate migration in those studies might be due to patient group (COPD heterogeneity), the selection of controls, or the assay utilized. In addition, the blood neutrophils function aberrantly in COPD patients with frequent exacerbation, including respiratory burst and phagocytosis (25).

Neutrophils migrate into the lungs by sensing and following chemo-attractant gradients of cytokines and bacterial peptides produced by tissues of infection or inflammation. Tasaka et al. found that among the 102 patients (mean age 68 years, 14 females), the levels of ENA78/CXCL5 and IL-8 are correlated with frequency or absolute numbers of neutrophils in BAL fluid (26). The migratory neutrophils release proteinase such as NE as they move through the extracellular matrix. In COPD patients, huge numbers of neutrophils migrate and accumulate in the lung and so large amounts of proteinases are released during this process, which might contribute to pulmonary damage by proteolysis of tissues. Furthermore, neutrophils recruited to the airway epithelium result in increased mucin production (27). Neutrophil serine proteases are the most potent inducers of mucin secretion although many stimuli induce secretion of mucin granules from epithelial cells. In addition, COPD sputa have large amounts of neutrophil extracellular traps (NETs) and NETotic neutrophils (28). However, the roles of the NETs in COPD have not been assessed. Taken together, neutrophils from COPD patients produce more NET and reactive oxygen species (ROS) with lower migratory accuracy and decreased phagocytosis.

Several drugs currently available for the treatment of COPD reduce mucus hyper-secretion in preclinical COPD models, but their effects on mucus hyper-secretion in humans have not been assessed. Testing the effects of these drugs on reducing mucus production and/or secretion will require performing specifically designed clinical trials. These trials will be necessary to explore the hypothesis that reducing mucus hyper-secretion is beneficial to the COPD patients (27). Recently, Walton and colleagues studied the effects of simvastatin (a lipid-lowering medication) on migratory dynamics of neutrophils in both COPD patients (mean age 69.5 years) and age-matched healthy donors (mean age 64.4 years) (15). Statins are used to help reduce cardiovascular risk, which is high in many COPD patients. They found that *in vitro* treatment of isolated peripheral blood neutrophils with simvastatin results in enhanced neutrophil velocity toward both IL-8 and fMLP (a potent polymorphonuclear leukocyte chemotactic factor) in COPD patients, restoring migratory accuracy to levels seen in control neutrophils from age-matched healthy donors (15). This finding suggests that statins may impact positively on some neutrophil functions, but further investigation is needed. NE inhibitor, AZD9668, was tested in a phase IIb trial (NCT00949975) in patients with symptomatic COPD (838 patients, males or post-menopausal females between 40 and 80 years old) receiving maintenance tiotropium, a long-acting anticholinergic bronchodilator. However, AZD9668 showed no effect on lung function, respiratory signs, and symptoms to those COPD patients (29), although NE induces the pathological characteristics of COPD *in vitro*.

IL-8 is one of the dominant neutrophil chemo-attractants in COPD lung secretion and CXCR2 is its chemoreceptor most engaged during neutrophil migration. Several CXCR2 antagonists have been tested in clinical trials. In a phase II trial with COPD patients (616 patients, mean 63 years, 438 males, FEV1% 47.8), treatment with 50 mg MK-7123 (CXCR2 inhibitor) versus placebo results in significant improvement in FEV1 in COPD patients, suggesting clinically important anti-inflammatory effects with CXCR2 antagonism, although dose-related discontinuations were observed because of absolute neutrophil number decreases with MK-7123 (30).

### Macrophage

COPD patients have innate immune dysfunction in the lung partly due to abnormal secretion of cytokines and chemokines by alveolar macrophages (AMs). Eapen et al. found that AMs show a dominant M2 phenotype in COPD subjects (31). BAL cytokines skew toward an M2 profile with increases in MDC/CCL22, IL-4, IL-13, and IL-10 in COPDs (31). Cigarette smoking is known to be the main common cause of COPD currently. Thus, cigarette smoke extract (CSE) is used to study its effects on AM function *in vitro*. Kent and colleagues found that CSE-treated macrophages from COPD patients (12 patients, all males, FEV1% 58) express reduced gene expression of many pro-inflammatory cytokines and chemokines (32). The exception of this pattern is that CSE treatment results in increased *IL-8* mRNA expression, which can attract neutrophils (32). In line with this, Metcalfe et al. found that CSE exposure suppresses TLR4-induced TNFα, IL-6, IL-10, but had no effect on IL-8 production at the protein level. The suppressing effect of CSE on LPS-induced cytokine production is correlated with a reduction in the activation of p38, ERK, and NF-κB signaling pathways (33, 34). Furthermore, AMs from COPD active smokers express higher levels of IL-8, but not TNFα, compared to those from COPD ex-smokers in response to respiratory pathogens, such as non-typeable Haemophilus influenza (NTHI), *Moraxella catarrhalis*, and *Streptococcus pneumonia*e (35). These findings suggest that COPD AMs are defective in cytokine responses under physiologically relevant conditions of exposure to both cigarette smoking and bacteria. AMs from exacerbation-prone COPD patients (29 patients, mean age 62 years, 21 males, FEV1% 57.6) are more refractory to TLR2 upregulation and cytokine induction by respiratory pathogens, compared with those from non-exacerbation-prone COPD patients (59 patients, mean age 58.1 years, 45 males, FEV1% 64.6) (36), indicating that COPD AMs with exacerbation have even more impaired function. Repetitive stimulation with the same TLR ligand induces TLR tolerance in COPD AMs, but this only occurs for selected cytokines or chemokines. For instance, IL-8 production is not reduced after repetitive stimulation with the same TLR ligand, which may be an important mechanism for the increased IL-8 observed in COPD patients (37).

Besides the abnormal secretion of cytokines and chemokines by COPD AMs, those AMs also express atypical chemokine receptors unable to trigger cell migration. For instance, the decoy chemokine receptor D6 is upregulated in AMs of 16 COPD patients with mean FEV1% 57, and its expression is correlated with the degree of functional impairment and the markers of immune activation (38). Increased expression of D6 in AMs could imply that D6 may have another roles in chronic inflammatory diseases possibly promoting immune activation besides its known scavenger activity in acute inflammation (38). Future studies on the mechanism underlying the contribution of AM-derived D6 to COPD pathogenesis are required.

Macrophage migration inhibitory factor (MIF) is a pleiotropic cytokine that antagonizes both apoptosis and premature senescence, which may be important in the pathogenesis of COPD. One study showed that both Mif(−/−) and Cd74(−/−) mice developed spontaneous emphysema compared with WT mice, which is associated with activation of the senescent pathway markers p53/21 and p16 (39). Following exposure to cigarette smoke, Mif(−/−) mice have more chances to develop COPD compared with WT mice, indicating that both MIF and the MIF receptor CD74 are required for maintenance of normal alveolar structure in mice. In contrast, She and colleagues found that MIF expression significantly increases in the smokers with COPD, and MIF level in the lung is positively correlated with airflow limitation, suggesting that MIF may play an important role in the pathogenesis of smoking-induced COPD (40). Therefore, careful translation of these murine findings into human COPD is urgently required.

COPD AMs are defective in their ability to phagocytose apoptotic cells (“efferocytosis”), and this defect is potentially linked to the sphingosine-1 phosphate (S1P) system, in particular, the sphingosine-1 phosphate receptor 5 (S1PR5) and antagonizing SIRP5 significantly improves phagocytosis (41). Barnawi et al. also found that S1PR5 is the single target that shows significant changes in DNA methylation between patient groups among the S1P system genes (42). AMs from COPD patients (20 patients, mean age 66.5, 14 males, FEV1% 76.5) show lower methylation levels in the same region compared to macrophages from non/ex-smokers. Reduced methylation may underlie the increased expression of the S1PR5 gene in AMs, which is associated defective efferocytosis in COPD (42). Furthermore, the impaired phagocytosis is also reported to be calcium-dependent processes. Extracellular calcium significantly improves phagocytosis in COPD monocyte-derived macrophages (43). Extracellular calcium increase expressions of bacterial recognition receptors, CD16, and macrophage receptor with collagenous structure (MARCO), while NTHI challenge leads to statistically significant reductions in CD16 (43). Moreover, specific calcium channel inhibitors abrogate calcium-mediated MARCO upregulation. These observations support the therapeutic use of calcium to improve macrophage function in COPD to decrease exacerbations and chronic bacterial infection. It may be necessary to test the effect of calcium on the SIPR5 expression, since both affect COPD AM efferocytosis.

In addition to macrophage dysfunction, COPD is associated with increased number of macrophages in the airways and lung. Blood monocytes leave the circulation and develop into AMs, which have the capacity to induce the pathological changes associated with COPD. Furthermore, monocytes from COPD patients (37 patients, 24 males, FEV1% 47) show increased chemotactic response toward growth-related oncogene (GRO) alpha/CXCL1 and neutrophil-activating peptide (NAP)-2/CXCL7, but not toward IL-8, when compared with controls (44). All these three chemokines bind to CXCR2 with high affinity, whereas IL-8 displays high affinity for both CXCR1 and CXCR2. CXCR2 expression on monocytes from COPD patients is regulated differently from nonsmokers and smokers in the presence of GROα, which may account for the enhanced migration toward GROα and NAP-2. This study suggests that increased recruitment of monocytes from the circulation may lead to increased macrophage numbers in COPD (44). The finding highlights the potential of CXCR2 antagonists as a therapy for COPD, since CXCR2 inhibitor may inhibit the chemotaxis of blood monocytes and neutrophils into the lung. Several CXCR2 inhibitors have been tested in clinical trials.

### Dendritic Cells

Dendritic cells play a role in the pathogenesis of COPD. In 2010, Galgani et al. detected a decreased number of blood plasmacytoid DCs (pDCs) and increased blood myeloid DCs (mDCs)/pDCs ratio in COPD patients (35 patients, mean age 62 years, 19 males, FEV1% 51.2) (45). Recently, one study showed that COPD blood pDCs display a decreased expression of the anti-inflammatory co-stimulatory molecule PD-L1 while COPD blood mDCs display an increased expression of the pro-inflammatory co-stimulatory molecule OX40 ligand (OX40L). The ratio of OX40L to PD-L1 expression is a quantitative measure of imbalanced DC co-stimulation, which is associated with the severity of pulmonary emphysema in patients with COPD (54 patients, mean age 59 years, 33 males, FEV1% 38) (46), suggesting that an imbalance of DC co-stimulation might contribute to the pathogenesis of COPD. In contrast to PD-L1, circulating pDCs in patients with COPD exposed to cigarette smoke express high levels of co-stimulatory molecules CD40 or CD86. *In vitro*, cigarette smoke directly promotes pDC maturation and release of IFNα, IL-6, and IL-12. These data suggested that circulating pDCs display an enhanced activation phenotype in patients with COPD (35 patients, mean age 62 years, 28 males, FEV1% 41.8) (47). In addition, blood pDCs from COPD patients express enhanced level of high-affinity IgE receptor (FcεRI), which is negatively correlated with the FEV1, the forced vital capacity and the maximum expiratory flow at 50% of the vital capacity. Patients with severe COPD (29 patients with GOLD III, mean age 65 years, 19 males, FEV% 38.3; 8 patients with GOLD IV, mean age 57 years, 5 males, EFV1% 22.6) and patients with allergic asthma show a similar FcεRI overexpression on pDCs. Moreover, the FcεRI expression on pDCs is positively correlated with total IgE serum concentrations (48). Considering the effect of anti-IgE on exacerbations in asthma, trials are urgently needed to test the effect of anti-IgE on exacerbations in severe COPD. Except OX40L, blood mDCs in COPD patients did not significantly differ from those in smokers with normal lung function in terms of expressions of BDCA-1, BDCA-3, CD86, and CCR5 (46), while monocyte-derived DCs of COPD patients display enhanced expressions of CD80 and CD86 (49), implying mature phenotypes.

Chemokine ligand 20 (MIP-3α/CCL20, the ligand for CCR6) is the most potent chemokine in attracting DCs. *CCL20* mRNA expression in human lung as well as CCL20 protein level in induced sputum are significantly increased in patients with COPD (10 GOLD I patients, mean age 60.8 years, 8 males, FEV1% 86.6; 16 GOLD II patients, mean age 66.6 years, 15 males, FEV1% 65.9; 10 GOLD III-IV patients, mean age 61.8 years, 6 males, FEV1% 39.3) compared with never-smokers (10 subjects, mean age 51.9 years, 4 males, FEV1% 98.6) and smokers without COPD (9 subjects, mean age 59.6 years, 7 males, FEV1% 102.5) (50). CCR6 at both mRNA and protein levels is detected in/on DCs isolated from human lung. Consequently, there is a significant increase in DC number in the epithelium and adventitia of small airway of COPD patients (50). Similarly, Su and colleagues detected increased S-100^+^ DC number in the lung of COPD patients compared to control subjects. S-100 serves as a marker to identify all DCs (51). These findings indicate that more circulating DCs infiltrate into the airways of COPD patients, which accumulate in the lung.

In terms of phenotype and function of lung DCs, Zanini et al. detected a decreased number of CD83^+^ cells and an increased CD207/CD83 ratio in bronchial biopsies from COPD patients, implying a reduced maturation of DCs (52). Moreover, CD83^+^ DCs are negatively correlated to VEGF and TGF-β expression in COPD patients (20 patients, mean age 76 years, 17 males, FEV1% 51) and the CD207/CD83 ratio is positively correlated to VEGF, TGF-β, and vascularity, suggesting that a reduced maturation of DCs in COPD is associated with airway vascularity and angiogenic factors. Similarly, Tsoumakidou and colleagues also found that there is a reduction in CD83^+^ mature DCs in COPD patients (28 patients, mean age 60 years, 26 males, FEV1% 64), both current and ex-smoker, compared to never-smokers and smokers after cessation, indicating that cigarette smoking and COPD *per se* are related with a decrease in pulmonary DC maturation (53). Additionally, immature DCs are significantly related to disease severity, since CD207^+^ cells are inversely related to FEV1. This finding implies that DC maturation may play a key role in the pathogenic mechanisms of COPD (52). Functionally, lung CD1c^+^ DCs from COPD patients hamper effector functions of T cells and favor the generation of suppressive IL-10-secreting CD4^+^ T cells that function through IL-10 and TGF-β in mixed leukocyte reactions. The levels of IL-27 and IL-10 are increased in the lung microenvironment on rhinovirus-induced COPD exacerbation *in vivo* (54). For the underlying mechanisms, Roghanian et al. observed that inflammatory lung secretion, NE, may account for the suppression of lung DC functions by interfering DC maturation and antigen-presenting activity (55).

In a clinical phase I trial, Lommatzsch et al. investigated the effects of fluticasone or fluticasone plus salmeterol on DCs in smokers with COPD GOLD 0 or 1 (56). Treatment with fluticasone plus salmeterol, but not fluticasone alone or placebo, results in a reduced endobronchial frequency of mDCs. In contrast, fluticasone reduces the frequency of pDCs, which is independently of salmeterol. No obvious changes in the expression of function-associated surface molecules on airway mDCs (such as CD1a, Langerin, BDCA-1, CD83, or CCR5) in all groups after treatment are detected. However, blood mDCs from smokers in the presence of Fluticasone (either alone or in combination with salmeterol) suppresses T-cell proliferation. Thus, resistance to fluticasone therapy in smokers might in part be due to lacking effects on airway mDCs, whereas the increased risk for infections during fluticasone therapy could be attributed to a reduction in pDCs. Combination therapy of fluticasone with salmeterol is related with a reduction in not only airway mDCs but also airway macrophages and neutrophils (56).

### Myeloid-Derived Suppressor Cells

MDSCs have been implicated in the regulation of chronic inflammation. Tan et al. found that the number of circulating MDSCs (CD14^−^HLA-DR^−^CD33^+^CD11b^+^ cells) is comparable in COPD patients with GOLD stages 2–4 and in control groups, regardless of COPD and smoking exposure. Depletion of MDSCs does not enhance activation and proliferation of CD4^+^ or CD8^+^ T cells, or alter production of IFNγ and IL-17 in response to staphylococcal enterotoxin B (SEB). However, depletion of MDSCs results in decreased TGF-β (57). By contrast, Sergio et al. observed that smoking exposure upregulates and activates circulating MDSCs (Lin^−^ HLA-DR^−^ CD33^+^ CD11b^+^ cells) both in smoker controls and COPD patients. And, this effect persists in COPD patients (53 patients, mean age 65.2 years, 43 males, FEV1% 61) after quitting smoking compared with smoker controls (58). This discrepancy could be due to the definition of MDSCs. Tan and colleagues detected monocytic MDSCs that express CD14, while Sergio and colleagues detected granulocytic MDSCs that display CD14 negative.

MDSCs downregulate the T cell receptor zeta chain (TCR zeta) through L-arginine deprivation and lead to T cell dysfunction. Different TCR zeta chain expression is significantly downregulated in circulating T lymphocytes of COPD patients as compared with both non-smokers and smokers (58). This finding supports the participation of MDSCs (Lin^−^ HLA-DR^−^ CD33^+^ CD11b^+^ cells) in the pathogenesis of COPD. Their recent study also showed that MDSC expansion was associated with TCR zeta downregualtion in COPD patients and TCR zeta down-regulation correlates with T cell hyporesponsiveness in COPD and lung cancer patients (59).

### Natural Killer Cells

Natural killer cells (NK cells) are reported to get involved in the pathogenesis COPD. Tang et al. detected increased numbers of NK (CD3^−^CD56^+^) and NKT-like cells (CD3^+^CD56^+^) in the peripheral blood in COPD patients (19 patients, mean age 55.84 years, 10 males, FEV1% 46.02) compared to healthy non-smokers (60). The frequencies of inducible IFNγ-secreting NK and NKT-like cells are less, while the frequencies of CD158a- and CD158b-expressing NK cells and CD158b-expressing NKT-like cells are higher in COPD patients. Furthermore, the frequency of CD158b^+^ NK cells is negatively correlated with several lung function parameters. This finding indicates that higher frequencies of inhibitory NK cells and NKT-like cells in the peripheral blood may participate in the pathogenesis of COPD (60).

Wang et al. investigated the activation levels of NK cells in both peripheral blood and induced sputum from healthy non-smokers, healthy smokers, ex-smokers with COPD, and current smokers with COPD (61). In the peripheral blood, current smokers regardless of disease state show the highest percentage of activated NK cells compared with ex-smokers with COPD and healthy non-smokers. In addition, NK cell activation is positively correlated with the number of cigarettes currently smoked. In contrast, the proportion of activated NK cells in induced sputum is significantly higher from both current smokers with COPD (5 patients, mean age 69 years, 2 males, FEV1% 59) and ex-smoker with COPD (6 patients, mean age 62 years, 4 males, FEV1% 59) compared to healthy non-smokers, which is related to disease state rather than current smoking status, suggesting that modulating NK activation may be a new target for the treatment of COPD (61).

### Eosinophil

A subset of patients with COPD demonstrates eosinophilic inflammation either in their sputum or blood. In those COPD patients, eosinophilia is related with an increased risk of 12-month COPD-related readmission, an increased risk of 12-month all-cause readmission, and a shorter time to first COPD-related readmission (62). These findings suggest the levels of peripheral eosinophil can be used as a biomarker in severe COPD exacerbations to predict higher readmission rates (62). In addition, COPD patients (10,861 patients) with circulating eosinophil counts of 2% or more of blood leukocytes respond better to inhaled corticosteroids than do those with counts of less than 2% (63). Several recent evidences suggest that blood eosinophil count is a promising biomarker of response to inhaled corticosteroids in patients with COPD (63, 64). Furthermore, patients with COPD with lower blood eosinophil counts of less than 2% have more pneumonia events than do those with higher eosinophil counts (63). All these indicate that eosinophil participates in COPD development, but the underlying mechanism remains largely unclear.

In a clinical phase III trial, IL-5 inhibitor, Mepolizumab, is testing its effects on eosinophil number (https://clinicalTrials.gov identifier: NCT02105961), since sputum eosinophil levels are elevated to similar levels as those seen in severe asthmatics. It is hypothesized that the reduction of eosinophils with mepolizumab in COPD subjects would translate into reduction of COPD exacerbations.

### Adaptive Immune System

Long-term exposure to cigarette smoke causes local inflammation in the airways that involves not only innate immune cells but also adaptive immune cells such as T cells and B cells.

### CD8^+^ T Cells

COPD patients have increased circulating frequency of CD8^+^ T cells, while healthy smokers have higher percentage of CD8^+^ regulatory T cells (CD8^+^ Tregs). Increased CD8^+^ T cells are inversely correlated with declined FEV1 in COPD (65). In terms of CD8^+^ T cell subsets, the frequency of Tc1 cells is increased in both stable COPD (SCOPD) and acute exacerbation COPD (AECOPD) patients, whereas the percentage of Tc2 cells is decreased in SCOPD patients but remains normal in AECOPD patients (66). The percentage of Tc17 cells is increased only in AECOPD patients, and the percentage of Tc10 cells is reduced in both SCOPD and AECOPD patients. The imbalances of pro/anti-inflammatory Tc subsets observed in COPD may be caused by the lack of Tc10 cells and the impaired anti-inflammatory capacity of CD8^+^ Tregs. The imbalances also contribute to the immune response dysfunction in COPD pathogenesis (66). Mechanistically, Qiu et al. found that smoke-activated blood pDCs in COPD patients induce differentiation of IFNγ-expressing CD8^+^ T cells as well as IL-17-expressing CD8^+^ T cells from mouse naïve CD8^+^ T cells (47). In addition, blood CD8/CD28 (null) cells have been found to increase in both current (30 patients, mean age 59 years, FEV1% 57) and ex-smoker (18 patients, mean age 60 years, FEV1% 62) COPD groups, which express significantly higher levels of IFNγ, OX40, 4-1BB, CTLA4, granzyme, and perforin when stimulated than CD8/CD28^+^ T cells. Increased production of pro-inflammatory cytokines and expression of alternative co-stimulatory molecules by CD8/CD28 (null) T cells may play a role in inflammatory or autoimmune responses in COPD (67). Elevated numbers of Tc1 cells and their significant loss of the co-stimulatory molecule CD28 in COPD are consistent with findings in the elderly and in clinical conditions involving chronic activation of the immune system.

In the airways of patients with COPD, increased number of CD8^+^ T cells is also observed (68–71). Freeman and colleagues used resected lung from COPD patients (8 patients) to investigate the mechanisms for CD8^+^ T cell recruitment to the lung (72). They found that lung CD8^+^ T cells express CCR5 as well as CXCR3 and the expression levels are positively correlated with COPD severity. Lung CD1a^+^ DCs produce the respective ligands for CCR5 and CXCR3, MIP-1α/CCL3, and MIG/CXCL9, and the ligand levels are correlated with disease severity. Interestingly, a close proximity of CD8^+^ and CD1a^+^ cells in a bronchocentric process is observed, indicating pulmonary CD8^+^ T cell infiltration *via* CCL3-CCR5 and CXCL9-CXCR3 chemotactic ways. In addition to these two, COPD lung tissue is found to release more RANTES/CCL5, suggesting a role for CCL5-CCR3 signaling in the recruitment of pulmonary CD8^+^ T cells in COPD patients (15 patients, mean age 64.6 years, 4 males, FEV1% 64) (73).

In view of gender, CD8^+^ T cells in BAL express increased chemokine receptor CCR5 in female smokers with COPD compared to those without COPD, while CCR5 expression on CD4^+^ and CD8^+^ T cells in BAL is decreased in male smokers with COPD compared to those without COPD (74). Furthermore, higher frequency of CXCR3^+^ CD8^+^ T cells in blood is detected in female smokers with COPD compared to those without COPD (74). Among female smokers with COPD, Th1/Tc1 immune response is linked to BAL macrophage numbers and goblet cell density, while Th2/Tc2 response is associated with the measures of emphysema on high-resolution computed tomography. The highly gender-dependent T-cell profile in COPD indicated different links between cellular events and clinical manifestations in females compared to males. The findings may uncover mechanisms for the difference in clinical course in female COPD patients compared to male patients.

Lung CD8^+^ T cells in COPD are not thought to be the terminally differentiated effector populations. *In vitro* stimulation of lung CD8^+^ T cells with IL-18 and IL-12 results in more IFNγ and TNFα, whereas IL-15 stimulation induces an increase in perforin. Furthermore, both IL-15 and IL-18 proteins can be detected in whole lung tissue homogenates. These data indicate that the production of pro-inflammatory cytokines and cytotoxic molecules by lung CD8^+^ T cells contribute to COPD pathogenesis (75). Moreover, lung CD8^+^ T cells in COPD express increase levels of surface TLR4 and TLR9. And those CD8^+^ T cells exposed to cigarette smoke condensate upregulate expressions of TLR4 as well as TLR9 and increase cytokine production (76).

IL-17A^+^CD8^+^ T cells are detected in lung biopsies of patients with COPD by immunohistochemistry (77). Chang and colleagues found that IL-17F is significantly higher in the bronchial biopsies from COPD patients (16 patients, mean age 53 years, 10 males, GOLD I 2 patients, II 8 patients, and III–IV 6 patients) compared to control subjects. In the submucosa, the absolute number of both IL-17A and IL-17F positive cells is increased in COPD patients. The expressions of IL-17A and IL-17F are co-localized with not only CD4 but also CD8, which are further confirmed at RNA level (77). These findings support the notion that Tc17 cells are involved in the pathogenesis of COPD.

The percentage of lung PD-1-expressing CD8^+^ T cells is greater in COPD patients (33 patients, mean age 67 years, 16 males, FEV1% 76.13) than control subjects (78). Infection by influenza virus leads to PD-1 upregulation on CD8^+^ T cells from both controls and COPD patients. However, influenza infection results in significant upregulation of the marker of cytotoxic degranulation (CD107a) on CD8^+^ T cells from control subjects, but not those from COPD patients, suggesting that the dysfunction of PD-1^+^CD8^+^ T cells in COPD may also play a role in the pathogenesis of COPD (78).

### CD4^+^ T Cells

#### Th17 Cells

Th17 cells are the key component of systemic inflammation in COPD. IL-17A and IL-17F enhance innate immune responses, especially affecting the development of neutrophil airway inflammation. COPD patients have an increase of blood Th17 cells and the presence of blood Th17 cells is negatively associated with FEV1% (79). In small airways, the number of IL-17A-expressing inflammatory cells in the subepithelium is increased in COPD patients (18 patients, mean age 72 years, 13 males, FEV1% 63.5) compared to that in smokers and non-smokers (80). The level of IL-17A is higher than that of IL-17F in this region, while IL-17F expression is greater than IL-17A in epithelial cells and lymphoid follicles, implying different roles of IL-17A and IL-17F in the pathogenesis of COPD. IL-17A contributes to small airway subepithelial inflammation, whereas IL-17F might play a more prominent role within lymphoid follicles (80). Moreover, the number of Th17 cells in the small airways is positively correlated with airflow limitations (81). Increased Th17 cell number in lung tissue (small airways and alveolar walls) plays a role in chronic inflammation of the lungs and parallel lung injury aggravation in COPD patients (10 patients, mean age 62 years, all males, FEV1% 55) and in smokers without airway limitations (81).

Th17 cells and Th17-polarizing cytokines partially drive airway inflammation. Statin could suppress IL-17 cytokines in some diseases such as multiple sclerosis in mouse model. NCT01944176 is a clinical phase III trial, which investigates whether statins might suppress IL-17 to provide anti-inflammatory benefit in SCOPD patients without an exacerbation.

#### Treg Cells

In 2003, COPD was hypothesized as an autoimmune disease for autoantibodies develop in some COPD patients. The increasing recognition that COPD shares features with autoimmune diseases has led to interest in a potential role for Treg cells, which are intimately involved in the control of autoimmunity. Human Treg cells consist of three distinct subsets: CD25^++^CD45RA^+^ resting Treg cells (rTregs), CD25^+++^CD45RA^−^ activated Tregs (aTregs), which are suppressive, and CD25^++^ CD45RA^−^ cytokine-secreting (Fr III) cells with pro-inflammatory capacity (82). Hou et al. compared the frequency of Treg subpopulations in smokers and COPD patients (14 patients, mean age 72 years, 8 males, FEV1% 45) (83). In blood, increased proportions of rTregs, aTregs, and FrIII cells are observed in smokers compared with never-smokers, whereas COPD patients show a reduction in rTregs as well as aTregs and a significant increase in FrIII cells compared with smokers. The changes in Treg subpopulations in COPD patients, with an overall decrease in the ratio of (aTreg + rTreg)/Fr III, indicate that immune homeostasis favors inflammation. The functional defects in Treg cells may further enhance inflammation, which contribute to the pathogenesis of COPD. Moreover, depletion of Treg cells in PBMCs from 15/15 healthy controls and 9/14 COPD patients increases SEB-induced activation of conventional CD4^+^ T cells. Impaired Treg-mediated suppression of CD4^+^ T cell activation is detected in a subset of COPD patients, all of who had high body-mass index (83). Therefore, obesity and/or perturbed homeostasis of Treg subsets may explain this defect and so contribute to increased inflammation observed in COPD.

In the BAL samples, the majority of Treg cells lacks the expression of CD45RA. The percentage of aTregs decreases whereas Fr III cells increase in patients with COPD compared with control groups. Furthermore, the ratios are significantly decreased in BAL of COPD patients, indicating that disturbed Treg homeostasis is also present in the lung. Another study performed by Smyth et al. found that Treg cells in the BAL samples of patients with COPD display weaker functional regulatory ability (84). More interestingly, the ratios of (aTreg + rTreg)/Fr III in the BAL from COPD patients (66 patients, mean age 68 years, 56 males, FEV1% 47) show more robust correlations with FEV1% predicted value and activation of effector T cells than those in the blood (85). These findings suggest that the imbalance between the anti-inflammatory subpopulations (aTreg + rTreg) and the pro-inflammatory subpopulation (FrIII) of Treg cells may play an important role in the pathogenesis of COPD.

#### Th17/Treg Ratio

The imbalance of Th17/Treg cells plays an important role in the pathogenesis of COPD. Wang and colleagues showed that significantly increased blood Th17 cells and Th17-associated cytokines and transcriptional factor, but decreased blood Treg cells and Treg-associated cytokines and transcriptional factor are observed in patients with moderate (32 patients, mean age 66 years, 16 males, FEV1% 53.4) and severe COPD (33 patients, mean age 68 years, 28 males, FEV1% 36.3) (86). Furthermore, the increased ratios of Th17 to Treg cells are inversely related with lung functions. In sputa, the level of *FOXP3* mRNA is lower while the level of *RORC2* mRNA is markedly higher in COPD patients. There is a correspondingly elevated ratio of sputum *RORC2* to *FOXP3* mRNA in COPD. Thus, a compartmental imbalance of Th17 over Treg cells exists in the airways of patients with COPD, suggesting a defect in anti-inflammatory homeostasis in COPD (87).

In addition to smoke-related COPD, in those COPD patients who were exposed to sulfur mustard (SM-exposed COPD patients), *FOXP3* expression is lower and *RORC* expression is higher in lung biopsies (88). The relative *FOXP3* expression and *FOXP3/RORC* ratio are positively correlated with lung function. However, the relative *RORC* expression is inversely correlated with FEV1 and relative *Foxp3* expression. IL-10 is significantly decreased in SM-exposed COPD patients at both RNA level and protein level. These findings suggest that the imbalance of Treg cells over Th17 cells plays a role in the progression of chronic lung injury of SM-exposed COPD patients (88).

The mechanisms underlying the imbalance of Th17/Treg cells still remain largely understood in patients with COPD. Plasma caveolin1 is one of those factors. The treatment of Caveolin1 siRNA is accompanied by an augmentation of Treg and reduction of Th17 expression in a study with patients with SCOPD (mean age 62 years, 25 males, FEV1% 45.72) and acutely exacerbated COPD (mean age 60 years, 23 males, FEV1% 31.11). Plasma Caveolin1 loss contributes to the imbalance of Th17/Treg cells (89). Furthermore, Zhang et al. found that the increased plasma BAMBI levels in COPD patients (29 patients, mean age 58.9 years, 25 males, FEV1% 51.4) positively correlate with the enhanced plasma TGF-β1 levels and Th17/Treg ratio (90). This finding suggests that an impaired TGF-β/BAMBI pathway may enhance the inflammation leading to Th17/Treg imbalance (90).

### B Cells

More recently, several reports demonstrated that B cells have strongly linked to COPD. COPD is characterized by an excessive activation of the adaptive immune system and, in particular, uncontrolled expansion of the B-cell pool. Polverino and colleagues found an increase in B cell counts as well as an increase in the number and size of B cell-rich lymphoid follicles in COPD lungs that correlated directly with COPD severity (91). Lymphoid follicles from COPD lung express the homeostatic chemokine BLC/CXCL13 and CXCL13 is the most important inducer of lymphoid follicles in COPD lungs. Litsiou et al. showed that lung B cells from patients with COPD (32 patients, mean age 65 years, 24 males, FEV1% 72.5) are important sources of CXCL13 and lymphotoxin and also expressed their receptors (92). Furthermore, CSE-LPS exposure upregulate B cell-derived CXCL13. The LPS-induced increase in CXCL13 is partly mediated *via* lymphotoxin. Notably, CXCL13 is required for efficient B-cell migration toward COPD lung homogenates and induces lung B cells to upregulate lymphotoxin, which further promote CXCL13 production, establishing a positive feedback loop (92). Therefore, blockade of CXCL13 in cigarette smoke-exposed mice leads to decreased number of organized lymphoid follicles, attenuation of airway inflammation, and partial protection of mice from alveolar destruction (93). In addition to CXCL13, pulmonary lymphoid follicles of patients with end-stage COPD express SDF1/CXCL12, which may increase proliferation and survival of B cells to promote COPD severity (94).

B cell products such as autoantibodies directed against lung cells, components of cells, and extracellular matrix proteins are also detected in COPD lungs. Active smoking induces relatively high levels of memory B cells in blood and in the lung. More switching to IgG is observed in COPD smokers (12 patients, mean age 67 years, FEV1% 65), while more switching to IgA is observed in healthy smokers (11 subjects, mean age 52 years, FEV1% 109) (95). Stimulation with lung-specific antigens results in higher numbers of anti-decorin antibody-producing cells in COPD patients compared with healthy controls. Differential switching to IgG and IgA suggests that the adaptive immune response to smoke differs between COPD patients and healthy controls. A higher percentage of memory B cells in the lungs of COPD patients may show an antigen-specific immune response and the antigen could be decorin in COPD lung (95). These autoantibodies may get involved in lung inflammation and injury in COPD patients partially by forming immune complexes that activate complement components.

An increase in mediators in lung that promote B cell maturation, activation, and survival in COPD patients is observed. A proliferation-inducing ligand (APRIL) is one of the key promoters of B cell expansion. The frequency of APRIL-expressing B cells and AMs is higher in the lungs of patients with both COPD and NSCLC than in patients with COPD or NSCLC alone or control subjects (96). BAFF is another mediator for B cell survival, maturation, and differentiation. BAFF has been linked to COPD, since COPD patients were reported to have increased BAFF expression in bronchial epithelium (97). In addition, BAFF levels are higher in blood and BAL B cells in patients with COPD (19 patients, mean age 56 years, FEV1% 46) compared with controls (98). Taken together, these mediators promote the progression of COPD.

The drug for treatment for COPD, fluticasone reduces IgG expression at both protein and mRNA levels and downregulates B cell proliferation through the dephosphorylation of ERK-1/2 *via* increasing the expression of dual-specificity protein phosphatase 1 (99). Consistent with *in vivo* data, oxidative stress failed to prevent fluticasone-induced suppression of IgG expression in human B cells *in vitro*. Thus, fluticasone may reduce adaptive immune response in COPD (99).

Thus, both human COPD studies and studies of murine COPD models support the notion that B cells play a role in COPD pathogenesis and especially the emphysema phenotype. However, a clinical trial of rituximab, a monoclonal antibody against CD20 that is expressed on pre-B cells and matured B cells, but not on other cells, for the treatment of COPD was terminated early due to increased pulmonary infection rates in the rituximab arm (100). This trial suggests that COPD is a complex immune disorder that too many immune cell subsets get involved in. Targeting one single subset of immune cells is not enough for disease-modifying therapeutics for COPD patients.

## Conclusion

**Figure 1:**
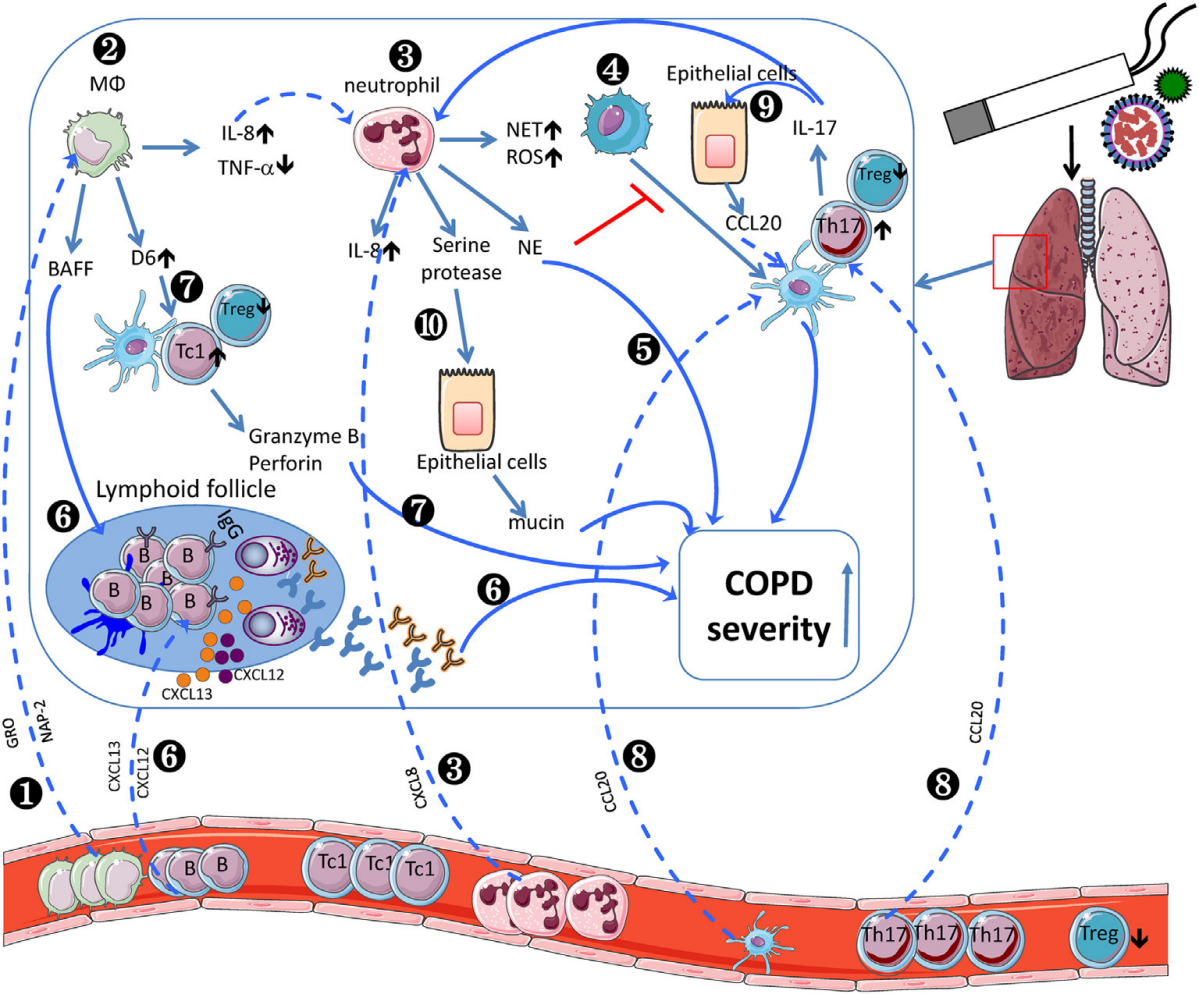
Inflammatory cells involved in the pathogenesis of chronic obstructive pulmonary disease (COPD). COPD monocytes leave the circulation, migrate into the lung, and differentiate into M2-like macrophages by sensing growth-related oncogene (GRO)/CXCL1 and neutrophil-activating peptide (NAP)-2/CXCL7 (❶). In lung, cigarette smoke extract (CSE) induces alveolar macrophages (AMs) to produce IL-8/CXCL8 (❷), which recruits circulating neutrophils (❸). Neutrophils produce neutrophil elastase to inhibit dendritic cell (DC) maturation (❹) and decline pulmonary function (❺). Moreover, AM-induced BAFF could promote B cell development and lymphoid follicle maintenance (❻), which recruit peripheral B cells *via* BLC/CXCL13 or SDF1/CXCR12. Activated B cells produce a large amount of antibodies (❻), leading to exacerbated COPD severity. AM-expressing D6 promotes granzyme B and perforin-expressing TC1 response, which exacerbate COPD severity (❼). In another aspect, COPD epithelial cells secret MIP-3α/CCL20, resulting in increased recruitment of dendritic cells and Th17 (❽). IL-17F further promotes lung epithelial cells to secret CCL20 (❾). Neutrophil-derived serine protease induces epithelial cells to secrete more mucin to exacerbate COPD severity (❿). The crosstalk between adaptive and innate immune cells contributes to the pathogenesis of COPD.

The role of innate and adaptive immune system in COPD pathogenesis still remains largely unknown. Current evidences indicate that a wide variety of immune cell subsets participate in COPD progression (Table 1). And the crosstalk between innate and adaptive immune cells leads to increased COPD severity (Figure 1). CSE induces AMs to produce IL-8, which recruits circulating neutrophils to the lung. Neutrophils produce NE to make damage to the lung during migration as well as inhibit DC maturation. Moreover, AM-induced BAFF could promote B cell development and lymphoid follicle maintenance. Activated B cells produce a large amount of antibodies, leading to lung injury. AM-expressing D6 promotes TC1 response. In another aspect, COPD epithelial cells secret CCL20, resulting in increased recruitment of DCs and Th17. IL-17F further promote lung epithelial cells to secret CCL20. Taken together, the crosstalks among those immune cells contribute to the pathogenesis of COPD.

**Table 1:** Immune cells and chronic obstructive pulmonary disease (COPD).

Immune system		Blood			Lung		
		Cell frequency/number	Cell phenotype	Correlation with COPD severity	Cell frequency/number	Cell phenotype	Correlation with COPD severity
Innate	Neutrophil	Increase	Poor migratory accuracy	Positive	Increase	Neutrophil elastase, NET, reactive oxygen species, IL-8	Positive

	Alveolar macrophages	–	–	–	Increase	M2	Positive

	Dendritic cells (DCs)		Myeloid DCs: OX40L ↑	–	Increase	Immature	Positive

		pDCs ↓	Plasmacytoid DCs: CD86 and FceRI ↑				

	Myeloid-derived suppressor cells	gMDSCs ↑	Activated	–	–	–	–

	NK cells	Inhibitory	CD158a, CD158b	–	Activated NK ↑	CD69, CD25	Positive

		NK ↑					

	Eosinophil	Increase	–	Positive	–	–	–

Adaptive	CD8	Increase	Tc1	Positive	Increase	CCR5, CXCR3, PD-1	Positive

	Th17	Increase		Positive	Increase		Positive

	Treg cells	Fr III ↑		Negative	Fr III ↑		
		rTreg ↓	Fr III cells		rTreg ↓	Fr III cells	Negative

		aTreg ↓			aTreg ↓		

	B cells	–	Memory B	Positive	Increase	IgG-B cells	Positive

On the other hand, each immune cell type may contribute to the disease pathogenesis at different stage. For instance, B cells are unlikely to be involved in the initiation and/or progression of early stage human emphysema, as greater declines in FEV1 occur in mild COPD patients in whom pulmonary B cell counts are lower and lymphoid follicles are small and infrequent when compared with those in advanced disease.

So far, there are still knowledge gaps on those immune cell subsets that contribute to COPD pathogenesis. Current evidence supports the notion that lung DCs display reduced maturation and impaired antigen-presenting capacity, which favor the induction of Treg cells. However, there is decrease in the frequency of Treg cells in COPD lung and increase in the frequency of Tc1 cells. Future studies will be done to address this inconsistency. Only by fully understanding of their respective roles in the onset and progression of COPD as well as their crosstalk can we design some potential disease-modifying drugs for the patients with COPD.

## Author Contributions

LN and CD wrote this review article.

## Conflict of Interest Statement

The authors declare that the research was conducted in the absence of any commercial or financial relationships that could be construed as a potential conflict of interest.
